# A deep learning architecture for metabolic pathway prediction

**DOI:** 10.1093/bioinformatics/btae359

**Published:** 2024-07-29

**Authors:** Mayank Baranwal, Abram Magner, Paolo Elvati, Jacob Saldinger, Angela Violi, Alfred O Hero

**Affiliations:** Department of Electrical Engineering and Computer Science, University of Michigan, Ann Arbor, MI 48109, USA; Department of Computer Science, University at Albany, SUNY, Albany, NY 12222, USA; Department of Mechanical Engineering; Department of Mechanical Engineering; Department of Mechanical Engineering; Department of Chemical Engineering and Biophysics, University of Michigan, Ann Arbor, MI 48109, USA; Department of Electrical Engineering and Computer Science, University of Michigan, Ann Arbor, MI 48109, USA

## Abstract

**Motivation:**

Understanding the mechanisms and structural mappings between molecules and pathway classes are critical for design of reaction predictors for synthesizing new molecules. This article studies the problem of prediction of classes of metabolic pathways (series of chemical reactions occurring within a cell) in which a given biochemical compound participates. We apply a hybrid machine learning approach consisting of graph convolutional networks used to extract molecular shape features as input to a random forest classifier. In contrast to previously applied machine learning methods for this problem, our framework automatically extracts relevant shape features directly from input SMILES representations, which are atom-bond specifications of chemical structures composing the molecules.

**Results:**

Our method is capable of correctly predicting the respective metabolic pathway class of 95.16% of tested compounds, whereas competing methods only achieve an accuracy of 84.92% or less. Furthermore, our framework extends to the task of classification of compounds having mixed membership in multiple pathway classes. Our prediction accuracy for this multi-label task is 95.62%. We analyze the relative importance of various global physicochemical features to the pathway class prediction problem and show that simple linear/logistic regression models can predict the values of these global features from the shape features extracted using our framework.

**Availability and implementation:**

https://github.com/baranwa2/MetabolicPathwayPrediction.

## 1 Introduction

Metabolic pathways are comprised of a linked series of chemical reactions occurring within a cell, where chemical products from one reaction act as substrates for the next reaction. The substrates in each pathway are catalyzed into structurally similar products by catalytic enzymes. Understanding the mechanisms and structural mappings between molecules and pathway classes are critical for design of reaction predictors for synthesizing new molecules (Pireddu *et al.* 2006; Sankar *et al.* 2017) or optimizing drug metabolization (Cho *et al.* 2010). Knowledge of metabolic pathways can also elucidate compound toxicity mechanisms (Nicholson *et al.* 2002). The primary focus of this article is to develop and assess a high-fidelity model that, given a chemical structure representation of a molecule, can accurately predict its pathway class associations.

A number of approaches have been employed for correlating protein annotations to pathway templates in order to derive organism-specific pathways. These range from data retrieval strategies (Boudellioua *et al.* 2016; Covell 2017; Hamdalla *et al.* 2015) to machine learning methods (Dale *et al.* 2010; Fang and Chen 2017; Khosraviani *et al.* 2015; Moore *et al.* 2019; Wang *et al.* 2017; Zelezniak *et al.* 2018), molecular fragments representation (Chen *et al.* 2016) and network integration methods (Guo *et al.* 2018). As a result, several popular tools for analyzing metabolic pathways have appeared in the literature, including PathComp (Kanehisa *et al.* 2006), PathPred (Moriya *et al.* 2010), Pathway Tools (Karp *et al.* 2009), UM-BBD Pathway Prediction System (Ellis *et al.* 2008), MRE biosynthesis pathway finding tool (Kuwahara *et al.* 2016) and TrackSM (Hamdalla *et al.* 2015). Several methods have been developed specifically for the problem of classification of compounds into metabolic pathway classes. These have been validated on publicly available metabolic pathway databases. These databases include the Kyoto Encyclopedia of Genes and Genomes (KEGG) database (Kanehisa and Goto 2000), EcoCyc/MetaCyc database (Karp *et al.* 2000), Expert Protein Analysis System (ExPASy) database (Gasteiger *et al.* 2003), Cell-Signaling Networks Database (CSNDB; Takai-Igarashi *et al.* 1998), PathDB (Mendes *et al.* 2000), UM-BBD (Ellis *et al.* 2008) and Signaling Pathway Database (SPAD; Tateishi *et al.* 1995). Among them, KEGG is often used for benchmarking classification performance of pathway prediction methods. KEGG is a manually curated database of pathway maps consisting of links to specific information about compounds, enzymes and genes. Several pathways in KEGG are characterized by the chemical structures of their main compounds, such as carbohydrates, lipids, polyketides and amino acids. Molecules are represented with names, chemical and structural formulas, metabolic pathways in which the molecules occur and enzymes that catalyze reactions containing the molecules.

In Cai *et al.* (2008), the authors proposed a nearest-neighbor (NN) algorithm to map small molecules to pathway classes by utilizing the functional group composition of these molecules. A set of 2764 compounds, with each compound belonging exclusively to one of the 11 identified pathway classes, was retrieved from the KEGG database for analysis. The authors obtained an overall accuracy of 73.3% for the NN predictor of metabolic pathway classes. The approach of Cai *et al.* (2008) is not directly extendable to compounds belonging to more than one pathway class. In Macchiarulo *et al.* (2009), the authors used a random forest (RF) classifier on 32 physicochemical and topological descriptors to predict association of 681 molecules with seven manually identified KEGG pathway classes and obtained an average Matthews correlation coefficient of 0.73.

Hu *et al.* (2011) proposed a multi-class model for predicting association of a query compound to one or more of the previously identified KEGG pathway classes. For the single-class prediction task, i.e. predicting compounds belonging to only one pathway class, they obtained an overall average accuracy of 77.97% using 5-fold cross-validation on a benchmark dataset consisting of 3137 compounds. Gao *et al.* (2012) further extended the work by Hu *et al.* (2011) and obtained an average prediction accuracy of 77.12% on a dataset comprised of 3348 small molecules using leave-one-out cross-validation (LOOCV) study. A major drawback with both these approaches is that they require knowledge about interactions between compounds in the dataset. As a result, the authors in Hu *et al.* (2011) and Gao *et al.* (2012) could not process 1229 small molecules due to the lack of sufficient interaction information.

Hamdalla *et al.* (2015) overcame the above limitation by finding scaffolds (substructures) that are shared commonly among structurally similar compounds. They hypothesized that compounds that share common scaffolds are associated with biochemically related pathways. A tool (TrackSM) was developed to extract scaffolds from compounds belonging to the same KEGG pathway classes and an average accuracy of 84.92% was obtained on 3190 small molecules using LOOCV. For a query compound with previously unknown metabolic pathway class, its scaffolds are matched against scaffolds of the compounds with known pathway associations, and the classifier declares the query compound to be a member of the class with largest match score.

In the past few decades, there has been significant growth in biomolecular databases (Dunn and Ellis 2005; Fiehn 2002). However, the use of these databases for predicting properties of novel biomolecular compounds remains a major challenge. To this end, machine learning (and deep learning in particular) has been applied to a variety of computational chemistry applications, including drug discovery (Sliwoski *et al.* 2014), toxicity prediction (Mayr *et al.* 2016), genomic prediction (Menden *et al.* 2013), protein–protein interaction prediction (Shoemaker and Panchenko 2007; Zhang *et al.* 2019), enzymatic function prediction (Li *et al.* 2017), biological reaction energy prediction (Alazmi *et al.* 2018), quantitative structure activity relationship (QSAR) modeling (Goh *et al.* 2017) and predicting the outcome of biological assays (Ma *et al.* 2015). In many such applications, the input data (e.g. chemical compounds) are highly structured, and so there is potential for specialized machine learning methods to extract relevant shape features more effectively than general purpose deep neural networks (Tsubaki *et al.* 2018). Extensions of these methods have additionally been used for the generation and optimization of chemical structures (You *et al.* 2018).

In this article, we propose a graph convolutional network (GCN) approach to classify query compounds into metabolic pathway classes and to determine discriminating features. The primary contributions of this article are summarized as follows:

- *Hybrid deep learning and ensemble learning approach*: A combination of a GCN-based deep learning architecture for graph representation learning and an RF classifier is proposed to predict the set of pathway classes to which a query compound may belong. A feature of the proposed architecture is that our prediction engine only requires the chemical structure (SMILES string) of the query compound. From this, it is able to perform the classification with an accuracy that is statistically significantly better than that of methods that are instead given access to global molecular features [such as Molecular ACCess System (MACCS) keys, molecular weight, water-octanol partition coefficient, etc.].
- *Multi-class classification*: Unlike existing methods on pathway prediction that do not naturally extend to multi-class classification, our architecture extends to mixed-membership classification of compounds into multiple pathway classes. To this end, we have suitably modified the original GCN architecture to account for multi-class classification.
- *General framework*: While the primary focus of this work is to predict the set of pathway classes for a query compound, the proposed GCN architecture can be easily applied for prediction of other metabolic properties, such as log P, toxicity and enzymatic functions.
- *Feature importance analysis*: The relative importance of 173 global molecular features is quantified, and their ranking is produced based on their discriminative capability. The analysis provides insights into features that contribute the most to distinguishing pathway classes. We find that the values of the top-ranked features for a given molecule can be predicted using the shape features generated by our GCN architecture, indicating the promise of the GCN approach as a data-driven substitute for laborious expert-driven feature engineering in chemical classification applications.
- *Interpretability of learned features in terms of chemical graph parameters*: In the Supplementary Material, we provide a methodology by which shape features learned by the GCN architecture can be observed to be tied, at least statistically, to chemical graph parameters, such as diameter. This hints that the problem of classification of compounds into pathway classes is related to global graph structural features of the molecules.

## 2 Materials and methods

Among the publicly available biological pathway databases, one of the most commonly used pathway database is the KEGG database (Kanehisa and Goto 2000). The KEGG database consists of 11 manually curated pathway maps that represent molecular interaction and reaction networks. These pathway classes are strongly correlated to biological functions of molecules. A total of 4935 compounds belong to one or several of these 11 identified metabolic pathway classes: Carbohydrate metabolism, energy metabolism, lipid metabolism, nucleotide metabolism, amino acid metabolism, metabolism of other amino acids, glycan biosynthesis and metabolism, metabolism of cofactors and vitamins, metabolism of terpenoids and polyketides, biosynthesis of other secondary metabolites and xenobiotics biodegradation and metabolism. Each of these 11 pathway classes further consists of several individual pathways.

A dataset of 4935 compounds belonging to one or more of these 11 constituent pathway classes was downloaded (February 2019) from the KEGG database: https://www.genome.jp/kegg/pathway.html. Of these 4935 compounds, a total of 4539 compounds belong to only one constituent metabolic pathway. Most prior work on predicting pathway classes focuses primarily on predicting pathway classes only for compounds belonging to a single class. While this approach greatly simplifies the overall prediction task, this viewpoint provides only partial information on biological functions of the remaining 396 compounds. Our work builds upon the single-class prediction solution and extends it to multi-class classification, where the objective is to identify all constituent pathway classes to which a compound may possibly belong. Thus, the approach prescribed in our work goes beyond the existing work on metabolic pathway prediction. Supplementary Figure S1 shows the distribution of compounds across 11 constituent pathway classes in the KEGG database.

### 2.1 GCNs for classifying molecular graphs

**Figure 1.**
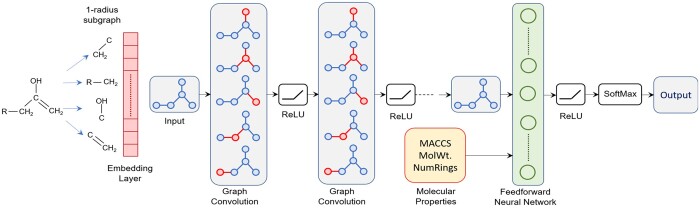
Proposed graph convolutional network for metabolic pathway prediction.

We propose a multi-layer GCN-based architecture for metabolic pathway prediction (summarized in Fig. 1). The GCN outputs a single probability distribution over classes in case of single-class prediction, while it outputs a vector of class membership probabilities in case of mixed-membership prediction. The input to the architecture consists of a graph *G* representing the molecule to be classified, along with a vector $\vec{w}$ of curated properties of the molecule. These properties include molecular fingerprints and the number of aromatic rings. The nodes of *G* correspond to atoms, and the edges *G* correspond to bonds between the atoms. Each node is labeled with its atom type, and each edge is weighted by the multiplicity of its bond.

The trained architecture works as follows: *G* is passed through the GCN, which results in a graph embedding vector ${\vec{v}}_{G}∊R^{d}$, where the embedding dimension *d* is a hyperparameter. Then, we concatenate ${\vec{v}}_{G}$ and a vector $\vec{w}$ consisting of the global molecular features of the molecule, resulting in a combined feature vector ${\vec{v}}_{\mathrm{emb}}$. This is passed through a feed-forward discriminative neural network, which is fully connected. The output of the network is a vector of class prediction probabilities summing to 1.

The embedding portion of the GCN works as follows: to each node *u* of *G*, we associate an initial *d*-dimensional feature vector, which encodes the *r-radius subgraph*—the subgraph induced by all nodes within *r* hops of *u* (for a hyperparameter *r*) as a vector (Tsubaki *et al.* 2018). This is in contrast to explicit inclusion of atom and bond features as likely feature vectors in Coley *et al.* (2019). In particular, each distinct possible *r*-radius subgraph is assigned a random unit-norm vector. Each layer of the GCN updates all node embedding vectors by first replacing each vector with the average over all neighboring vectors. This is followed by a linear transformation given by the trained model parameters. Each coordinate of the result is then passed through a *rectified linear unit* activation function. Finally, after a number of layers given by another hyperparameter, all of the final node embedding vectors are averaged, resulting in a *d*-dimensional graph embedding vector. In essence, the aggregation step of each successive layer stores increasingly coarse information about the graph in the node embedding vectors. The influence of a given level of coarseness is governed by the magnitudes of the weights corresponding to the given layer. The model parameters include the weight matrices of the GCN and of the fully connected feed-forward network. They are trained together by minimizing the standard cross-entropy objective function over the training set (Goodfellow *et al.* 2016). The use of the feed-forward network at the output allows for this to be done via stochastic gradient descent. The computation of gradients is done via backpropagation. The GCN architecture was implemented similarly to Kipf and Welling (2017). More precisely, given an input graph *G* with adjacency matrix *A* consisting of *N* nodes (atoms), and quantity $X^{\left(0\right)}∊R^{N\times d}$ representing the *d*-dimensional embedding of the nodes, an *l*-layer GCN updates node embeddings using the following transition function:

(1) $$X^{\left(t+1\right)}=\mathrm{ReLU}\left(\tilde{A}X^{\left(t\right)}W^{\left(t\right)}\right),\;\;\text{for}\;\text{all}\;t∊\{0,1,\ldots ,l-1\},$$

where $\tilde{A}={\hat{D}}^{-\frac{1}{2}}\hat{A}{\hat{D}}^{-\frac{1}{2}}$ is the normalized adjacency matrix. Here, $\hat{A}=A+I$ and $\hat{D}$ is the degree matrix of $\hat{A}$. Parameters $W^{\left(t\right)}∊R^{d\times d}$ denote the weight-matrix of the *t*th-layer of the GCN. The embedding $X^{\left(l\right)}$ generated by the final layer of GCN is averaged across its nodes to produce the graph embedding vector ${\vec{v}}_{G}$ given by:

(2) $${\vec{v}}_{G}=\frac{1}{N}{\left(\sum_{n=1}^{N}X^{\left(l\right)}[n,:]\right)}^{T}.$$

The graph embedding vector is concatenated with vector of global molecular features $\vec{w}$ to produce a combined feature vector ${\vec{v}}_{\mathrm{emb}}$, which is then passed through a neural network represented by f(·) to produce an 11-dimensional output vector $z≜f({\vec{v}}_{\mathrm{emb}})$. A final SoftMax layer (Goodfellow *et al.* 2016) is applied to produce a probability vector *y_out_*, which sums up to 1, i.e.

(3) $$y_{\mathrm{out}}=\mathrm{SoftMax}\left(f(\mathrm{concat}[{\vec{v}}_{G}\;\vec{w}])\right).$$

The training produces the following:

- A chemical structure feature extraction component, which takes chemical structures and outputs structural feature vectors relevant to the classification problem. We will also refer to these structural feature vectors as GCN embeddings. This component is extracted from the layers prior to the feed-forward network.
- A classification component, which takes as input the extracted structural features and global molecular features and yields class membership probabilities.

After training, the structural feature extraction component can be used to generate input features to train ensemble classifiers, such as an RF classifier (Breiman 2001). RF aggregates outputs from multiple decision tree classifiers to decide the final class (label) of the query object. This results in a classification accuracy that is better than competing methods. This is in contrast to the architecture in Tsubaki *et al.* (2018), which does not use ensemble methods and instead only considers a feed-forward neural network for the classification component of the problem that it considers.

## 3 Experiments

Six different machine learning models are compared for the prediction task: (i) RF classifier with local graph features, which takes as input the concatenation of the initial GCN node embedding vectors encoding the shapes of the two-radius subgraphs of the nodes; (ii) RF classifier with global molecular features, which takes 166-dimensional MACCS strings, as well as seven additional molecular descriptors as inputs. These additional descriptors are widely applied (Ghose *et al.* 1999; Lipinski *et al.* 1997; Oprea 2000; Veber *et al.* 2002) in drug discovery to determine bio-availability and activity of small molecule compounds due to their known influence on characteristics of molecules that affect their propensity to react in given settings, such as size (captured by molecular weight), rigidity [captured by rotatable bonds and ring counts (Lawson *et al.* 2018)], lipophilicity [captured by *logP* (Wildman and Crippen 1999) and aromaticity (Ritchie and Macdonald 2009)] and polarizability [captured by molar refractivity (Melville and Hirst 2007; Wildman and Crippen 1999)]. We refer to these 173 total features as global molecular features. (iii) RF classifier with GCN embeddings, which takes as input the output node embedding vectors (i.e. learned shape features) of the trained GCN; (iv) GCN which takes only chemical structure (via SMILES) as input; (v) GCN that takes chemical structure and global molecular features as input; (vi) GCN for multi-class classification, which takes SMILES and the global molecular features described above as inputs.

For both GCN and RF, the hyperparameters of the models are tuned in order to achieve the reported accuracies. In both cases, this tuning is done by performing a grid search over the set of possible hyperparameter settings. The parameters for the RF classifier include the number of base classifiers (300), maximum tree depth (60) and splitting criterion (Gini impurity). The hyperparameters of our GCN implementation are as follows: optimizer: Adam optimizer (Kingma and Ba 2014) with learning rate $\lambda =10^{-3}$; loss function: cross-entropy; number of epochs = 100; embedding dimension *d *=* *50; number of GCN layers *l *=* *3; and subgraph radius *r *=* *2. For the above choice of hyperparameters, the GCN comprises of nearly 8364 weights to be trained during the learning phase.

All models are implemented in Python 3.6.5 on an Intel i7-7700HQ central processing unit with 2.8 GHz x64-based processor. The SMILES are converted to a graph representation using the RDKit (Landrum *et al.* 2006) (version 2018.03.2). For RF classifier, we use the readily available implementation in the scikit-learn (Pedregosa *et al.* 2011) module (version 0.21.3), while our GCN is implemented in PyTorch (Ketkar 2017) (version 0.4.1).

### 3.1 Single-class classification

Of the 4539 KEGG compounds that belong to only one pathway class 3631 (80%) compounds are selected randomly for the purpose of training the models. The remaining 908(20%) compounds are split equally into cross-validation and test sets. The test examples are kept separate and the model performances are evaluated on the test set at the end of the training process. This process is repeated 10 times, and the mean statistics of these 10 runs are reported with randomly selected training, test and cross-validation sets. For each experiment, we report in Table 1 statistics for top *n* accuracy (*n *=* *1, 2, 3), where a classifier is said to have correctly characterized the pathway class for a query compound if the true class is among the top *n* classes predicted by the classifier. In below sections, we discuss the results of the application of the various classifiers to the test data.

**Table 1.** Performance analysis of several machine learning methods.

Methods	Accuracy score (%)		
	Top 1	Top 2	Top 3
Hu *et al.* (2011)	77.97	NA	NA
Cai *et al.* (2008)	73.30	NA	NA
Gao *et al.* (2012)	77	79	85
Hamdalla *et al.* (2015)	84.92	92.82	95.39
RF w/local graph features	21.47±1.0	39.96±1.5	59.76±1.5
RF w/global features	88.01±.47	95.05±.52	96.70±.69
RF w/GCN embeddings	**95.16**±**.68**	**98.20**±**.63**	**98.99**±**.54**
GCN	88.79±.95	93.49±.74	95.44±.98
GCN + global features	90.21±.92	94.73±.61	96.70±.72

*Note:* The differences between RF with global features, GCN and GCN plus global features were found to be statistically insignificant. The difference between these and RF with GCN embeddings was found to be statistically significant. NA, not applicable.

#### 3.1.1 RF classifier with local graph features

We apply the RF classifier to local graph features in order to compare the abilities of RF and the GCN architecture to extract graph structural feature information. In particular, the local graph features are the two-radius subgraphs of all nodes in the input graph. The performance of RF with local features is substantially worse than that of all other methods tested, indicating the inability of the RF classifier to extract relevant features directly from graph-structured information.

#### 3.1.2 RF classifier with global molecular features

With access to the global molecular features, the RF method significantly outperforms the other state-of-the-art methods with overall average accuracies of 88.01% (Top 1), 95.05% (Top 2) and 96.7% (Top 3), respectively. However, as we will show in subsequent sections, our GCN architecture, using only molecule structure as input, is capable of achieving the *same* performance *without* the need for careful hand-selection of features. Furthermore, a combined approach will be shown to yield even better performance.

#### 3.1.3 RF classifier with GCN embeddings

Here, we use the shape features extracted via the trained GCN as inputs to an RF classifier. To that end, we first train a GCN classifier to produce representations of molecules that can be easily distinguished by the feed-forward neural network at the output of GCN. We then use this trained GCN model to produce embedding vectors (at the output of last graph convolutional layer after activation) and feed them to an RF classifier. The intuition behind this hybridization is that a simple classifier is capable to learn complex functional relationship as long as the features provided to it are sufficiently rich. We find that this method achieves better performance than all competing methods. In particular, using McNemar’s test to compare RF with GCN embedding input and RF with global feature input, we find *P* values 0.0059, 0.0165, 0.0125 for the null hypotheses that the two classifiers have equivalent performance in terms of Top 1, Top 2 and Top 3 accuracies, respectively. This indicates the efficacy of the GCN as a method for extracting relevant structural features from graph representations of chemical compounds.

#### 3.1.4 GCN with chemical structure input

The difference in performance between the GCN (with feed-forward network output) and the RF classifier with global molecular features is not statistically significant (McNemar’s test, Dietterich 1998) with the null hypothesis that the accuracies of the two methods are the same yields *P* values of 0.9999, 0.3105 and 0.6291 for the Top 1, Top 2 and Top 3 classification tasks. Moreover, unlike the RF classifier, the GCN works with only SMILES as input and does not require additional global molecular features.

#### 3.1.5 GCNs with additional global molecular features

Upon inclusion of global molecular features, we find that the GCN (again, with feed-forward network output) is equivalent in terms of performance to the RF with global molecular feature input. McNemar’s test cannot reject the null hypothesis that the accuracies of RF and GCN + global molecular features are equal (the *P* values are 0.9999, 0.5716 and 0.7905).

### 3.2 Multi-class classification

We now discuss the task of classification of compounds into multiple pathway classes (i.e. the *multi-class* classification problem). To our knowledge, the existing works that categorize compounds into pathway classes do not directly address this task. Instead, for example, Hu *et al.* (2011) and Gao *et al.* (2012) produce rankings of pathway classes for a query compound, based on similarity to other compounds in the dataset. Such rankings may be converted to estimates of membership in multiple pathway classes by fixing a number k∊{1,…,11} and declaring that all of the Top *k* pathway classes contain the query compound, while none of the remaining classes do. Our approach to mixed-membership multi-class classification is fundamentally different. For each of the 11 identified pathway classes, our modified GCN-based model outputs a probability that captures the likelihood of the query compound belonging to the class. If the probability for a given class is at least half, then the compound is declared to be a member of the class.

We modify the output layer in our GCN model and replace the SoftMax layer with a layer of element-wise sigmoid activation functions (Zeng *et al.* 2018). Recall that the output of a sigmoid unit is restricted between 0 and 1 and, therefore, can be used to represent probabilities of association to pathway classes. The GCN is trained to minimize the sum of the binary cross-entropy losses at the sigmoid units. The performance of our multi-class GCN model is depicted in Table 2. For the multi-class classification problem, accuracy is defined as follows:

$$\text{Accuracy}=\sum_{i=1}^{N}\sum_{c=1}^{11}\frac{{\left(\text{Correct}\;\text{predictions}\right)}_{i,c}}{N\times 11}\times 100\%,$$

where ${\left(\text{Correct}\;\text{predictions}\right)}_{i,c}$ is 1 if the classifier correctly predicts the label for the *i*th compound for pathway class *c*, and 0 otherwise. Here, *N* represents the total number of compounds. In other words, the accuracy is the fraction of all correctly predicted associations between compounds and pathway classes. Performance of a classifier is not only measured by the overall average accuracy but also by the observed precision and recall. For a binary classifier, precision captures the positive predictive rate (i.e. the fraction of examples that are declared to be positive that actually are positive), whereas recall captures the sensitivity of a model (the fraction of examples that actually are positive that are declared to be positive). In order to evaluate precision and recall, we look at average classification/misclassification rate for each query compound. For instance, let us assume that a query compound is associated with 3 out of 11 pathway classes, described by the association bit-string ‘10100100000’, where ‘1’ at *i*th position indicates that the compound is associated with *i*th metabolic pathway class, while ‘0’ at *j*th position indicates that the compound does not belong to the *j*th pathway class. Let us further assume that our classifier predicts the association bit-string ‘10001100100’. Then the number of true positives (TPs), true negatives (TNs), false positives (FPs) and false negatives (FNs) in this example are 2, 6, 2 and 1, respectively. Here, TPs correspond to correct identification of Classes 1 and 6, while TNs correspond to correctly identified non-associations with Classes 2, 4, 7, 8, 10 and 11. This process is repeated for all the compounds in the test set and the cumulative statistics for TPs, TNs, FPs and FNs are used to evaluate precision and recall as:

$$\text{Precision}=\frac{\text{TP}}{\text{TP}+\text{FP}},\;\;\;\text{Recall}=\frac{\text{TP}}{\text{TP}+\text{FN}}.$$

We note a counterintuitive feature of accuracy, precision and recall as performance measures: accuracy may be high while, simultaneously, precision and recall may be low. This can happen if there are many negatives (i.e. compound-pathway class pairs for which the compound is not in the pathway class) and many TNs, but few positives. Thus, accuracy alone can be a misleading measure of performance of the different classifications methods.

**Table 2.** Performance analysis of multi-class classification.

Methods	Scores (%)		
	Accuracy	Precision	Recall
Hu *et al.* (2011)	94.64	77.97	67.83
k NN classifier	89.84±0.80	43.40±2.85	42.07±2.12
Ensemble logistic regression	89.20±0.34	14.07±2.27	13.06±2.23
Independent RFs	95.47±0.60	75.40±2.88	72.08±3.17
GCN + additional features	**95.62**±0.42	**81.09**±3.15	**79.30**±1.12

We evaluate the performance of the proposed GCN-based multi-class classifier against the described approach by Hu *et al.* (2011) with *k* set to maximize precision (i.e. *k *=* *1). Additionally, we compare with approaches based on the *k* nearest-neighbor (*k* NN) classifier (Keller *et al.* 1985), the ensemble logistic regression classifier with multiple base learners (Verma *et al.* 1887), and 11 RF classifiers trained separately to recognize each class. The inputs to these classifiers are the global molecular features associated with query compounds. As can be seen in Table 2, the proposed multi-class GCN classifier outperforms the classical machine learning approaches. Note that the GCN model does not use the MACCS bits as input features, but rather relies on input embeddings generated by the *r*-radii molecular subgraphs. Additionally, the top two performing methods, namely the multi-class GCN classifier and the independent RF classifier, are further evaluated based on the averaged bit-wise hamming loss and exact match scores. These are obtained as (**0.045, 0.717**) and (**0.045**, 0.648), respectively for the two classifiers (bold numbers indicate best performance).

**Figure 2.**
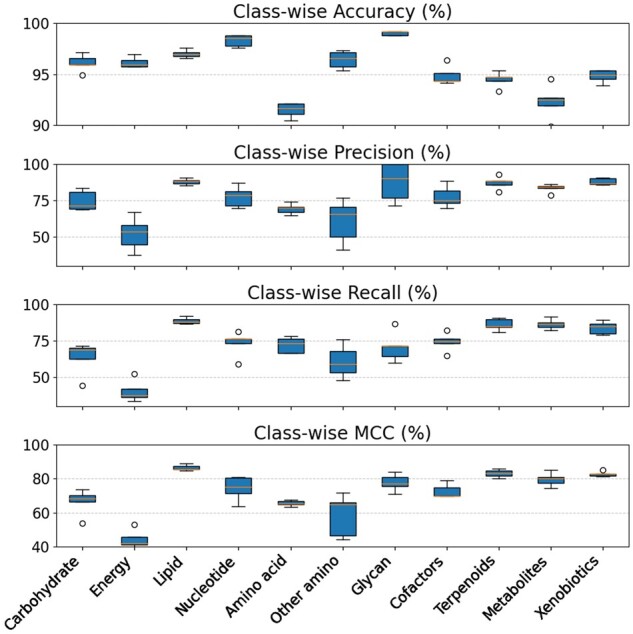
Class-wise performance statistics for the multi-class GCN classifier.

Our performance measures listed in the columns of Table 2 are useful as summaries of overall accuracy on the multi-class prediction problem. However, there remains a possibility that our classifier does poorly with respect to certain underrepresented pathway classes. In order to probe this possibility, we show in Figure 2 accuracy, precision, recall and MCC for our method on each individual pathway class. We see that there is no pathway class for which our method performs particularly poorly.

## 4 Discussion

Given the success of the GCN embedding approach in predicting pathway classes, it is of interest to understand better what these embeddings are capturing about the data. To this end, we performed an experiment in which we trained our architecture without the global molecular features, which yielded a trained GCN that could produce an embedding vector for each molecule in the dataset.

**Figure 3.**
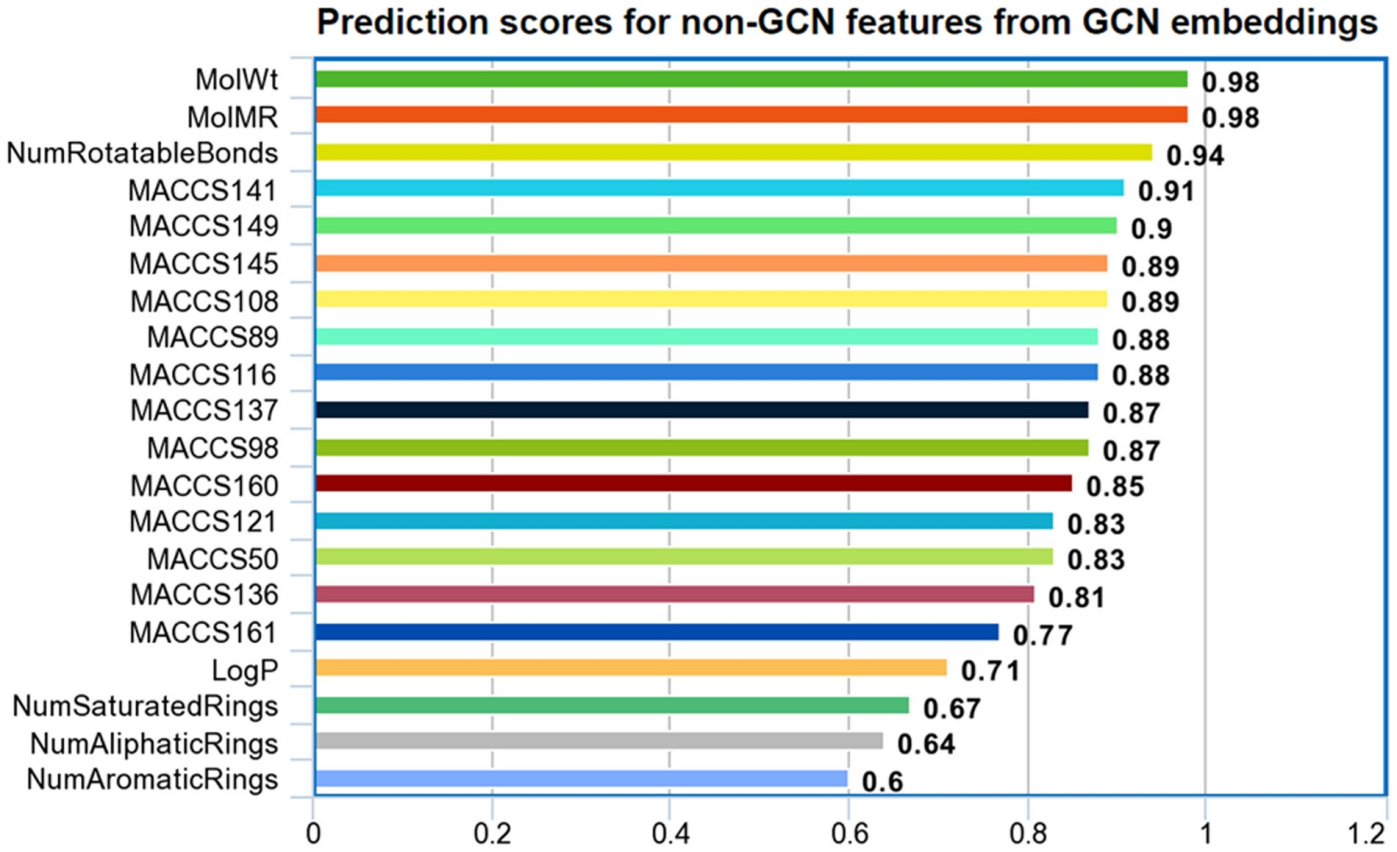
Prediction accuracy measures for regression models predicting global molecular feature values from GCN embeddings.

We then performed a linear/logistic regression analysis on the continuous-valued/binary-valued global molecular features, respectively, using the GCN embedding vectors as independent variables. In Figure 3, we give measures of the fit of these models for each of the most important global molecular features. For continuous-valued features, we give the adjusted *R*^2^ score, and for binary-valued ones, we give the empirical prediction accuracy (fraction of correct classifications) on a holdout set. These results indicate that the GCN embedding effectively captures the important global molecular features.

In addition to our exploration of the GCN embedding vectors in relation to global molecular features, we performed experiments to elucidate the interpretation of the embedding elements in terms of class-wise and purely graph-theoretic properties of the molecules. We also use *Shapley additive explanations* (Molnar 2019, Chapter 5.10) to estimate the average contribution of each feature to the classifier’s output in the presence of a uniformly random subset of other features. Detailed analysis of these experiments is included in the Supplementary Material.

## 5 Conclusion

This article proposes a GCN-based classifier to predict all metabolic pathway classes of which a query compound is a member. The experimental results demonstrate that a relatively low-dimensional feature embedding learned from graph structures, when used as input features to an RF classifier, outperforms classifiers based on global molecular features. Our GCN-based classifier achieves state-of-the-art performance on both single- and multi-class classification problems. Moreover, Shapley analysis of molecular descriptors provides insights into structural and physical properties that are relevant to determining associated pathway classes.

It is also worth noting that while GCN does not directly use molecular descriptors as input features, its output embeddings can be used to determine relevant molecular descriptors. This connection between the short-range connectivity and molecular properties is possible thanks to the somewhat limited type of atoms and bond patterns that commonly occur in biological molecules, which allow to characterize properties on local atomistic arrangements. For all the stems that share this locality, we conjecture that GCN embeddings retain relevant molecular information and can potentially be employed to develop novel molecular fingerprints in applications, such as drug design. Overall, the proposed framework is quite general and, while subject to availability of corresponding training data, the GCN-based framework can be made to learn and predict other useful molecular properties, such as toxicity and interaction with proteins.

## Supplementary data

Supplementary data are available at *Bioinformatics* online.

## Supplementary Material

btae359_Supplementary_Data
