# Supplementary material for: A deep learning architecture for metabolic pathway prediction
Source: Bioinformatics. 2024 Jul 29;40(7):btae359. doi: 10.1093/bioinformatics/btae359 (PMC11775948; doi:10.1093/bioinformatics/btae359)
Supplement: btae359_Supplementary_Data [file btae359_supplementary_data.pdf]

A deep learning architecture for metabolic pathway prediction

Mayank Baranwal, Abram Magner, Paolo Elvati, Jacob Saldinger, Angela Violi, and Alfred O. Hero

Supplementary Material

Dataset

A dataset of 4935 compounds belonging to one or more of these 11 constituent pathway classes was downloaded (February 2019) from the KEGG database: https://www.genome.jp/kegg/pathway.html. Figure 1 shows the distribution of compounds across 11 constituent pathway classes in KEGG database.

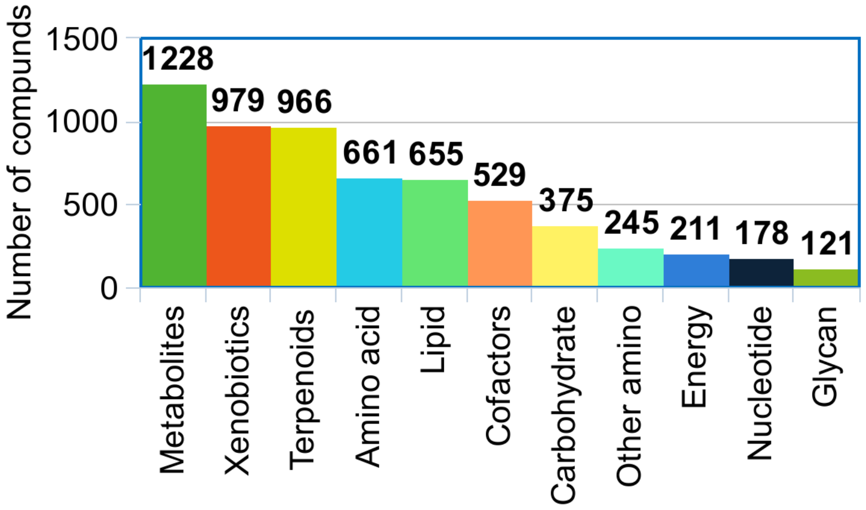

Figure 1: Distribution of 4935 compounds across multiple pathway classes in the KEGG database.

Feature Importance Analysis

We turn to the analysis of the relative importance of various features. The main findings are reported. First, the output of the GCN alone can achieve better performance than using only expert-chosen descriptors and MACCS fingerprints, so that the GCN architecture is a valuable addition to the chemical analysis toolbox. Second, our data-driven analysis of the relative importance of the expert-chosen descriptors and fingerprints (without the GCN output) is in agreement with previously reported experiments.

We use *Shapley additive explanations* (abbreviated *shap*) to estimate the average contribution of each feature to the classifiers output in the presence of a uniformly random subset of other features. The average contribution is quantified for that feature. Unlike other methods, e.g., greedy feature selection, the *shap* approach computes an average importance in the presence of random subsets of other features. Moreover, in its most basic form, *shap* is the *only* feature importance analysis method that satisfies natural axioms [3].

- Efficiency: The Shapley values for all features of a given data instance to be explained must sum to the difference between the value of the model output on that instance and the average value of the model output (taken over random instances from the data set). In other words, the difference between the prediction of a model on a given input and its average prediction is completely captured by the Shapley values of all of the features associated with that input.

- Symmetry: For a pair of features, if including either one or the other in the presence of an arbitrary set of other features results in the same model output, then the Shapley values of the two features are equal.

- Dummy: If a predictive model's output is constant with respect to a given feature, regardless of the inclusion or exclusion of other features, then the Shapley value of that feature is 0.

- Additivity: If a predictive model output is a linear combination $c_1x_1 + \cdots + c_kx_k$ of the outputs $\{x_i\}_{i=1}^k$ of k other models, then the Shapley value ϕ of a given feature for the combined model is given by $\phi = c_1\phi_1 + \cdots + c_k\phi_k$, where ϕ_i is the Shapley value for the given feature with respect to the ith model. For instance, the random forest classifier computes an average output of the outputs of its constituent decision trees. The Shapley value for a given feature for the classifier can thus be expressed as an average of the Shapley values of that feature for each of the decision trees.

We apply the Shapley method to rank the importance of features in terms of their contribution to the performance of the classifier in the following settings:

- random forest with global feature inputs,
- GCN with feedforward network output and global feature inputs,
- random forest with GCN embeddings and global feature inputs.

In the following paragraphs, for each setting we illustrate Shapley value estimates for the features in the multi-class setting via two diagrams: the first is a heatmap, where the intensity of the color at a given position indicates the Shapley value for the corresponding feature for predicting membership in the corresponding pathway class. The second gives cumulative information via a bar plot, where each feature has a corresponding bar, and the length of each bar gives the sum of the absolute Shapley values for that feature across all pathway classes.

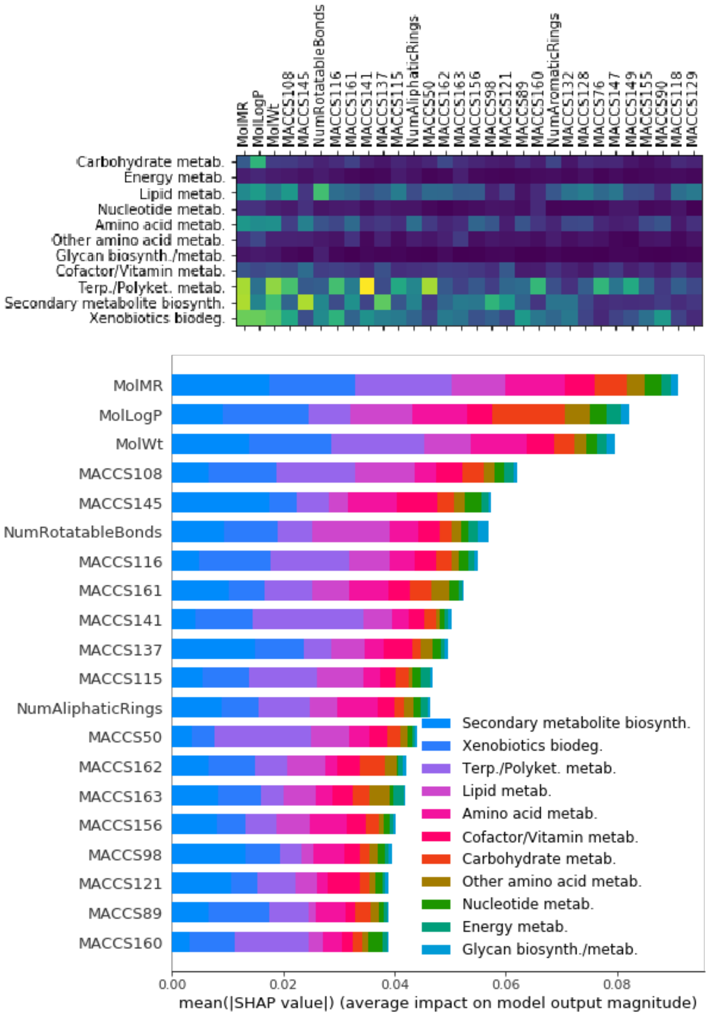

Figure 2: Top 20 Shapley values for Random Forest with global molecular features as input.

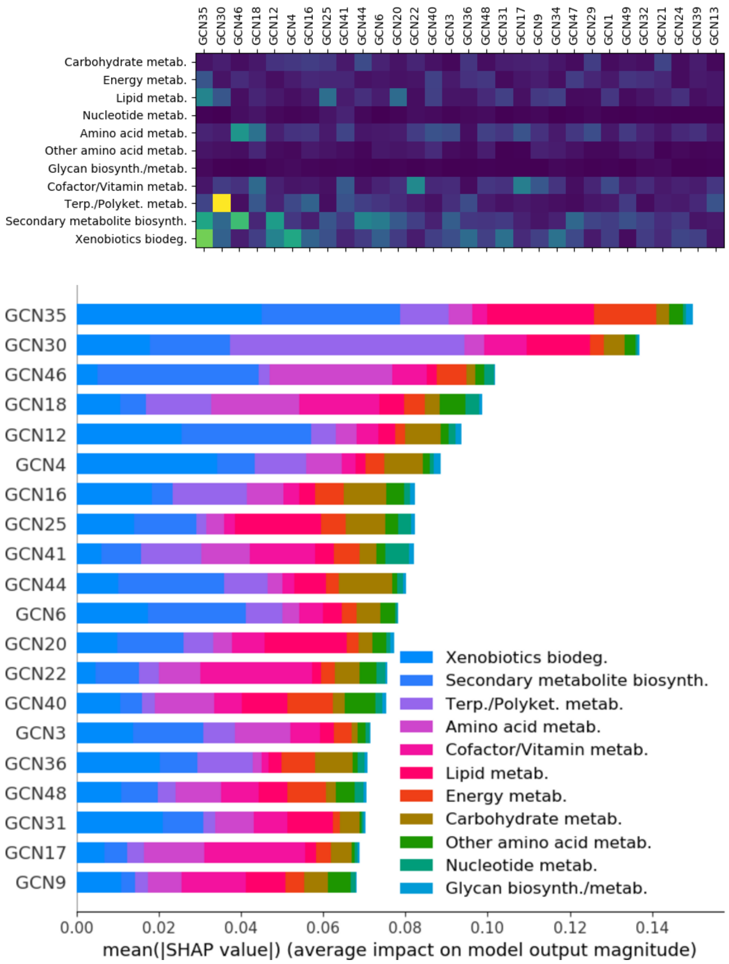

Figure 3: Top 20 Shapley values for GCN with global molecular features as input and feedforward network as output.

Feature Analysis For RF With Global Molecular Features.

In Figure 2, we plot Shapley value estimates for the top twenty global molecular in the multi-class setting. The features identified by the Shapley analysis correspond to factors that are known to influence small molecule metabolism. The most important identified feature is molar refractivity. This property correlates to the polarizability [13] of a molecule which has been shown to be an important property for predicting biological interactions [7] as it describes how a molecule's charge distribution is affected by an applied electric field such as in solution [1]. The second most important feature is the octanol-water partition coefficient, which is a measure of the molecule lipophilicity. This finding is in agreement with past studies [10,11] that have identified $\log P$ as one of the most important factors in distinguishing molecule interaction potency, due to its correlation with substrate transport and binding properties [8]. While $\log P$ is indicative of the molecule's propensity to reach and interact with the correct environment, it is not a specific measure for the interaction with a protein or enzymatic site. These interactions are primarily controlled by steric factors, and this general knowledge is matched by several of the top features, like the number of rotatable bonds, mass (as a rough estimator of the size), and several descriptors (e.g. MACCS 98, 121, 137, 145) related to rings, which are generally considered inflexible [5]. Beyond solubility and flexibility, the analysis indicates that other features are important, such as the presence of aromatic sites, as it promotes hydroxylation, which is key in many metabolic pathways [4,6], or the presence of methyl groups (MACCS 50, 108, 116, 141, 149, 160), which promote selectivity towards hydrophobic binding sites [12] and induce favorable binding conformations [2,9]. Finally, the presence of hydrogen bond donors and acceptors (MACCS 89, 136, 161) is the last group of features that was identified as relevant, in overall agreement with the work by Lipinski et al. [12].

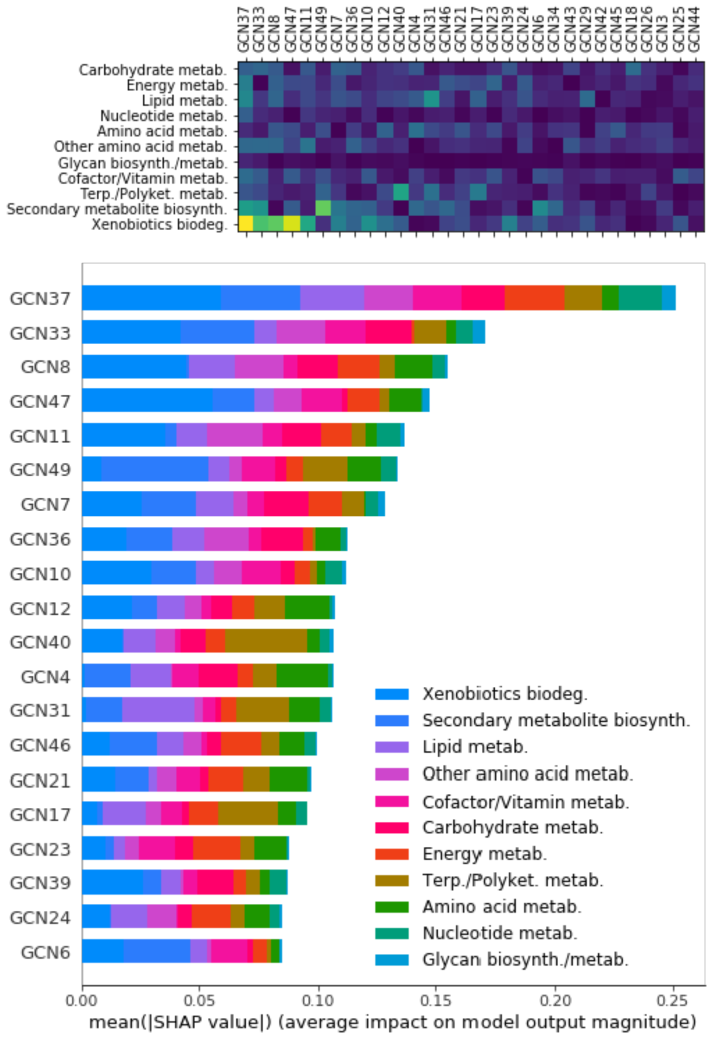

Figure 4: Top 20 Shapley values for Random Forest with GCN embeddings and global molecular features as input.

Feature Analysis For GCN With Global Feature Inputs And Feed-Forward Network Output.

In Figure 3, we give the Shapley plots for GCN with global feature inputs and feed-forward network output. We see, first, that the most important features for the prediction task, in a cumulative sense, are the GCN embedding features. Furthermore, for most pathway classes, there is no single feature that is overwhelmingly important. This indicates, first, that in the presence of GCN embeddings, global molecular features are treated as redundant by the feed-forward network, and, second, that information that is useful in distinguishing pathway classes is spread across various learned shape features.

Feature Analysis For RF With GCN Embeddings And Global Features.

In Figure 4, we give the Shapley plots for random forest with GCN embeddings and global features as input. We may draw similar conclusions from these plots as for those in the case of the GCN with feedforward network output and global feature input. However, we note that *different* GCN embedding features are important for the random forest output, in comparison to the feed-forward network output. We next delve more deeply into the analysis and interpretation of the GCN embedding features. Among other insights, we will give evidence that at least some GCN embedding elements that are highly ranked according to the cumulative Shapley analysis are capturing information about the same graph structural properties.

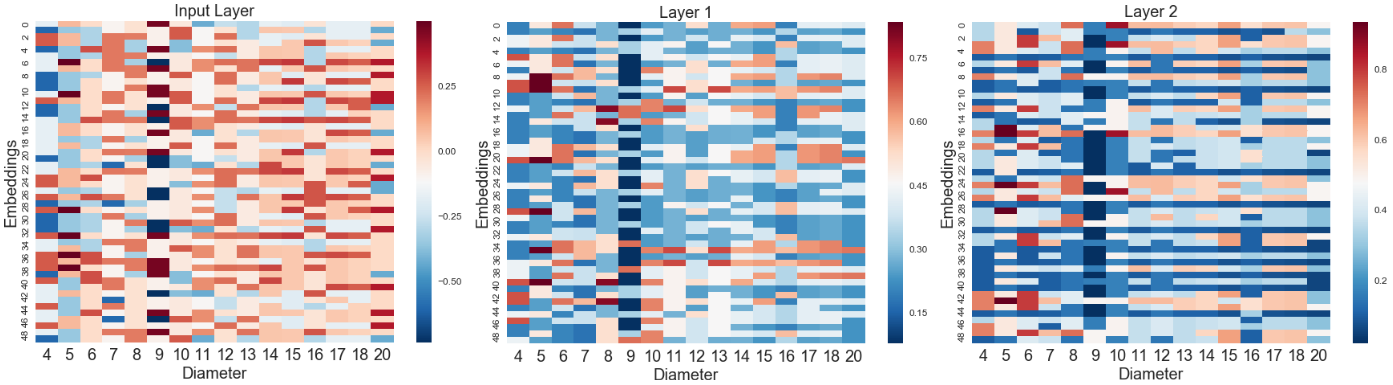

Figure 5: Layer-wise heatmaps of GCN embeddings of medoid molecules corresponding to different graph diameters.

Analysis Of GCN Embeddings

In addition to our exploration of the GCN embedding vectors in relation to global molecular features, we performed experiments to elucidate the interpretation of the embedding elements in terms of class-wise and purely graph-theoretic properties of the molecules.

The general pattern of the experiment was as follows: consider a graph property $f : G \to S$, where G is the space of molecular graphs, and S is an arbitrary range. For example, $f(G)$ may be the diameter of the graph G, in which case S is the set of non-negative integers. We partition the test dataset into the *level sets* of f, resulting in partition elements S_x, for each $x \in S$. When f is the diameter function, S_x is the medoid molecule in the test dataset with diameter exactly x.

Now, for each medoid molecule and each fixed GCN layer $l \in \{0, 1, 2\}$, we compute the GCN embedding vector $v_{G,l}$ of the graph after the lth layer. In general, the interpretation of this is as follows: as deeper layers of the GCN are explored, if there are many elements of embedding vectors that are correlated for each particular x, then this indicates a (potentially complicated) functional relationship between the activation of those embedding elements and the value $f(G)$. In such a case, we will say that the embedding elements are *sensitive* to f. We can visually depict the results of this experiment using layer-wise heatmaps (Figure 5) for the medoid molecules shown in Figure 9. Each of these medoid molecules has a different *graph diameter*. Each row in the heatmap corresponds to a given embedding element index, and each column corresponds to a diameter value. It can be observed that the embeddings corresponding to diameters 14, 15, 17 and 18 appear to be highly correlated. Interestingly, the medoid molecules corresponding to these diameter values are also structurally very similar, indicating a potential mapping of graphical structures to GCN embeddings. This experiment was repeated for other graph properties (radius and average degree), and a similar trend was observed (see Figure 8).

Figure 7 depicts sensitivity of few GCN embeddings in the final layer to graph diameters. It can be observed from that the embedding indices, such as, 3, 17, 25 and 30, which are also ranked highly by the Shapley analysis, appear to be sensitive to diameter. On the other hand, embedding indices, such as, 10, 28, 41 and 45, which do not contribute significantly towards classification task, appear to be insensitive to graph diameters.

We also perform a class-wise partition of the test dataset and depict the GCN embeddings corresponding to each of the 11 metabolic pathway classes averaged over all molecules belonging to the same class in Figure 6. Unlike in the previous experiment, no two classes seem to have significantly correlated embeddings in this case. This is critical from the point of view of classification problem, since molecules from different classes must have significantly different embedding vectors in order to be differentiated easily from one another. All of this gives evidence that the GCN embedding reflects information about global graph properties (such as the graph diameter) and that these play an important role in the classification of the molecules into pathway classes in both the feedforward neural network and random forest output cases.

Class-Wise Performance Of Multi-GCN Classifier

Table 1 (here) shows the class-wise confusion matrices for the multi-class GCN classifier evaluated on a held-out test set comprising of 987 molecules. Note that in multiclass tasks, labels are binarized under a one-vs-rest way, as

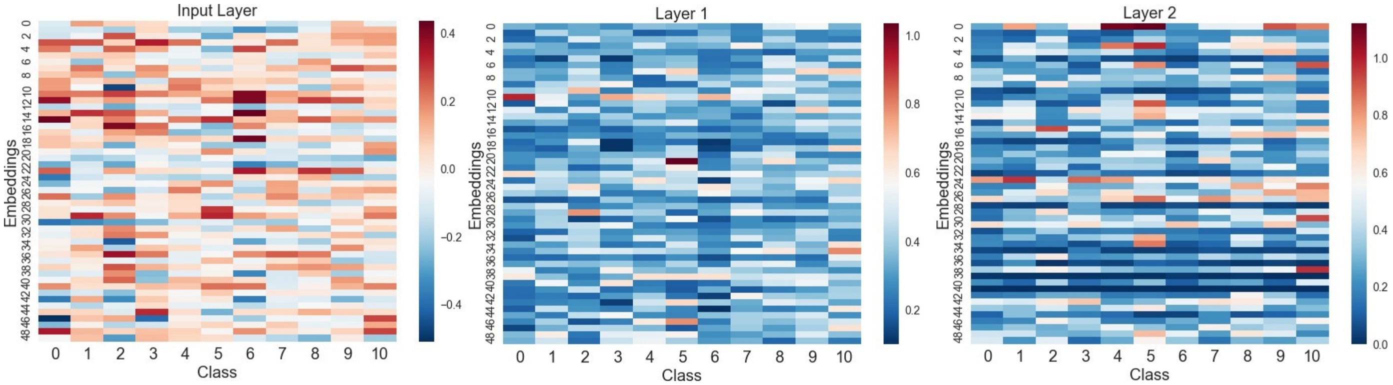

Figure 6: Layer-wise heatmaps of GCN embeddings of molecules belonging to different metabolic pathway classes. Note that there is sufficient variability among embeddings coming from two different classes, and thus GCN makes it easier for a classifier, such as, RF to be more effective at the classification task.

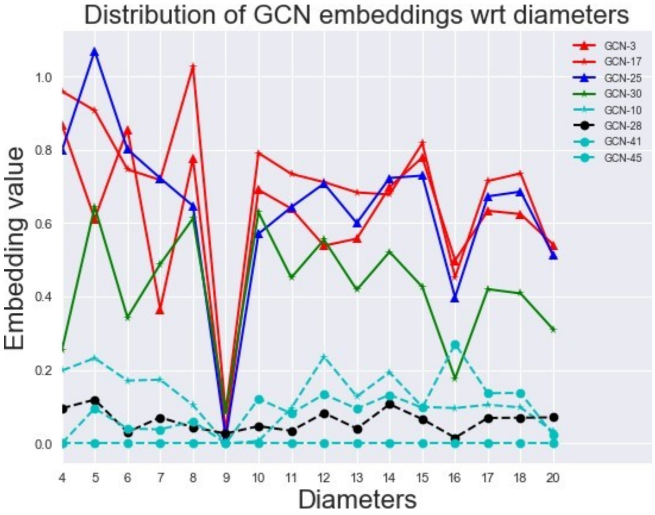

Figure 7: Sensitivity of selected GCN embeddings with respect to graph diameters. Features, such as, GCN-3, GCN-17, GCN-25 and GCN-30, which are identified as important by Shapley analysis, are sensitive to variation in graph diameters. On the other hand, features, such as, GCN-10, GCN-28, GCN-41 and GCN-45, which do not contribute significantly towards classification task, appear insensitive to variation in graph diameters.

in a mixed membership setup compounds can belong to multiple pathways. When calculating class-wise multilabel confusion matrix , the count of true negatives (TNs) for class i is $C_{i,0,0}$, false negatives (FNs) is $C_{i,1,0}$, true positives (TPs) is $C_{i,1,1}$ and false positives (FPs) is $C_{i,0,1}$.

Of these 987 molecules, there are 876 molecules which belong to only one pathway class. (1) provides the standard 11×11 confusion matrix corresponding to the 11 identified metabolic pathway classes.

$$\begin{bmatrix} 19 & 0 & 0 & 0 & 1 & 2 & 1 & 0 & 3 & 3 & 1 \\ 1 & 6 & 0 & 1 & 1 & 1 & 0 & 0 & 2 & 0 & 1 \\ 0 & 0 & 95 & 0 & 2 & 1 & 1 & 1 & 5 & 2 & 1 \\ 0 & 1 & 0 & 23 & 4 & 1 & 0 & 1 & 0 & 3 & 1 \\ 2 & 1 & 1 & 1 & 55 & 3 & 0 & 2 & 3 & 7 & 3 \\ 2 & 0 & 1 & 0 & 3 & 19 & 0 & 1 & 0 & 1 & 3 \\ 1 & 0 & 0 & 0 & 1 & 0 & 5 & 0 & 0 & 0 & 0 \\ 2 & 2 & 0 & 1 & 1 & 3 & 0 & 66 & 2 & 2 & 0 \\ 0 & 0 & 3 & 0 & 3 & 2 & 1 & 5 & 170 & 8 & 2 \\ 1 & 0 & 5 & 0 & 9 & 1 & 0 & 2 & 1 & 200 & 0 \\ 1 & 2 & 1 & 0 & 5 & 1 & 0 & 6 & 2 & 4 & 123 \end{bmatrix} \tag{1}$$

Additionally, the class-wise accuracy, precision, recall and MCC scores are shown in Table 2.

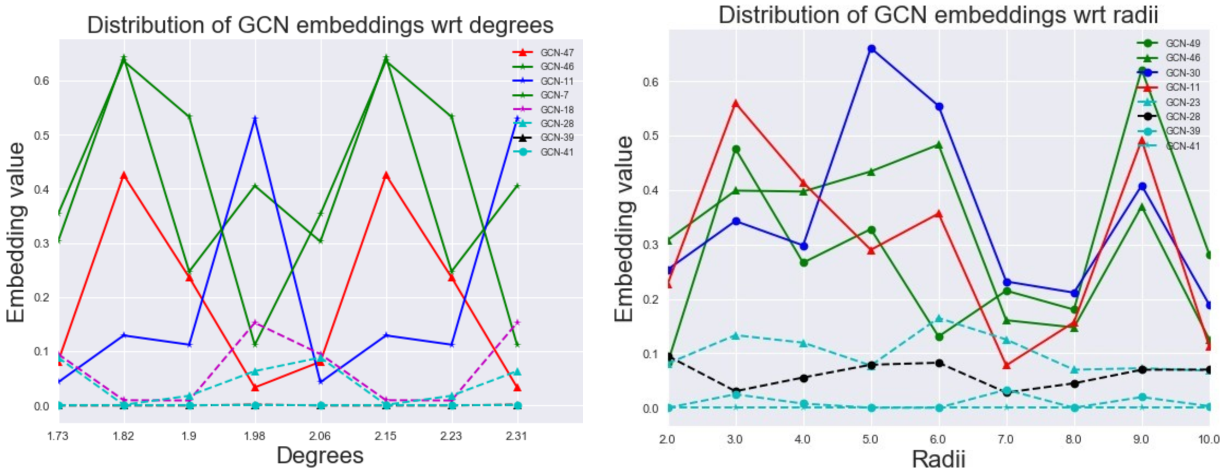

Figure 8: Sensitivity of selected GCN embeddings with respect to (a) average graph degrees, (b) graph radii. (a) Features, such as, GCN-47, GCN-48, GCN-11 and GCN-7, which are identified as important by Shapley analysis, are sensitive to variation in average graph degrees. On the other hand, features, such as, GCN-18, GCN-28, GCN-39 and GCN-41, which do not contribute significantly towards classification task, appear insensitive to variation in average graph degrees. (b) Features, such as, GCN-49, GCN-46, GCN-30 and GCN-11, which are identified as important by Shapley analysis, are sensitive to variation in graph radii. On the other hand, features, such as, GCN-23, GCN-28, GCN-39 and GCN-41, which do not contribute significantly towards classification task, appear insensitive to variation in graph radii.

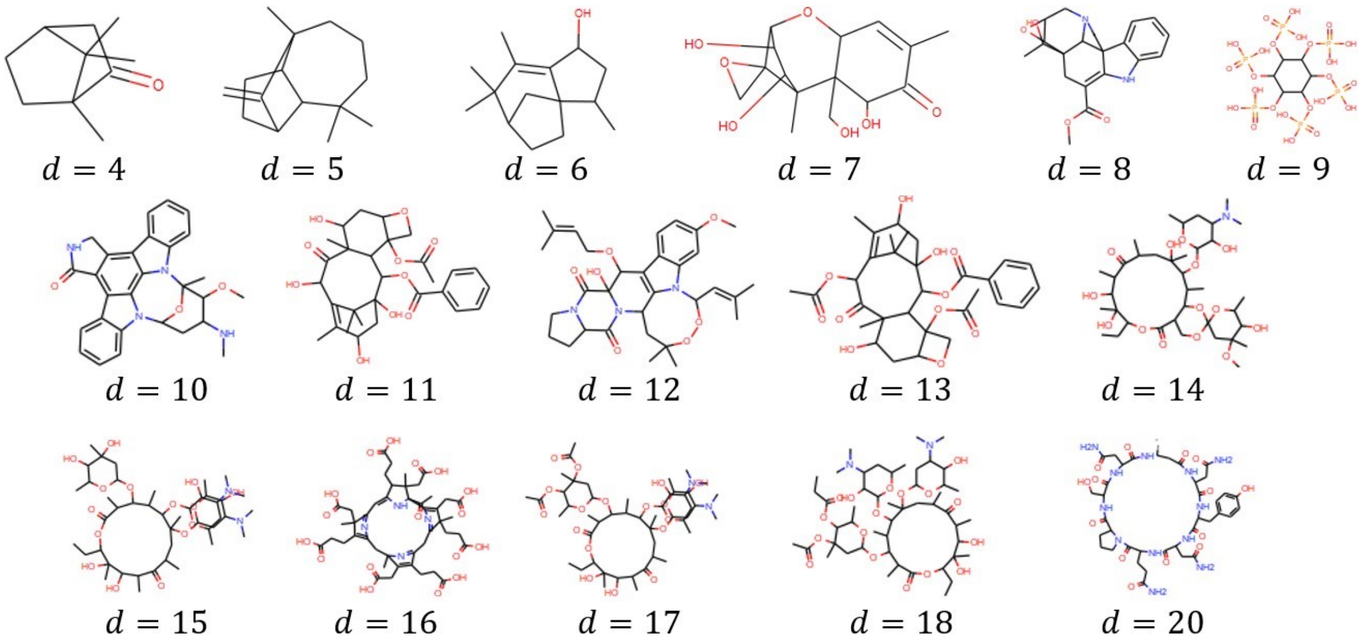

Figure 9: Medoid molecules corresponding to different graph diameter values: Note that the molecules corresponding to diameters 14, 15, 17 and 18 are structurally similar, and hence their GCN embeddings appear to be highly correlated in Figure 5.

References

[1] I. Banik and M. N. Roy. Study of solute–solvent interaction of some bio-active solutes prevailing in aqueous ascorbic acid solution. *Journal of Molecular Liquids*, 169:8–14, 2012.

[2] E. J. Barreiro, A. E. Kummerle, and C. A. Fraga. The methylation effect in medicinal chemistry. *Chemical*

Class	Scores
	Confusion Matrix
Carbohydrate	$\begin{bmatrix} 903 & 14 \\ 27 & 43 \end{bmatrix}$
Energy	$\begin{bmatrix} 936 & 12 \\ 22 & 17 \end{bmatrix}$
Lipid	$\begin{bmatrix} 851 & 17 \\ 10 & 109 \end{bmatrix}$
Nucleotide	$\begin{bmatrix} 941 & 8 \\ 9 & 29 \end{bmatrix}$
Amino acid	$\begin{bmatrix} 811 & 40 \\ 42 & 94 \end{bmatrix}$
Other amino acids	$\begin{bmatrix} 919 & 21 \\ 20 & 27 \end{bmatrix}$
Glycan	$\begin{bmatrix} 959 & 7 \\ 7 & 14 \end{bmatrix}$
Cofactors/Vitamins	$\begin{bmatrix} 860 & 23 \\ 26 & 78 \end{bmatrix}$
Terpenoids	$\begin{bmatrix} 749 & 25 \\ 32 & 181 \end{bmatrix}$
Sec. Metabolites	$\begin{bmatrix} 691 & 40 \\ 36 & 220 \end{bmatrix}$
Xenobiotics	$\begin{bmatrix} 802 & 13 \\ 28 & 144 \end{bmatrix}$

Table 1: Class-wise confusion matrix of multi-class GCN classifier

Class	Scores (%)			
	Accuracy	Precision	Recall	MCC
Carbohydrate	96.11 ± 0.74	74.65 ± 6.17	63.36 ± 10.09	66.50 ± 6.81
Energy	96.15 ± 0.46	51.97 ± 10.19	40.34 ± 6.65	43.48 ± 5.88
Lipid	97.00 ± 0.35	87.86 ± 1.84	88.51 ± 1.98	86.46 ± 1.55
Nucleotide	98.30 ± 0.52	77.50 ± 6.31	73.21 ± 7.63	74.37 ± 6.37
Amino acid	91.50 ± 0.63	69.16 ± 3.19	72.20 ± 4.79	65.64 ± 1.53
Other amino acids	96.44 ± 0.79	60.63 ± 13.19	60.77 ± 10.16	58.65 ± 11.07
Glycan	99.03 ± 0.20	87.58 ± 11.82	70.76 ± 9.07	77.72 ± 4.50
Cofactors/Vitamins	94.86 ± 0.83	77.46 ± 6.70	74.12 ± 5.65	72.77 ± 3.78
Terpenoids	94.49 ± 0.67	87.08 ± 3.96	86.14 ± 3.58	83.12 ± 2.04
Sec. Metabolites	92.31 ± 1.50	83.42 ± 2.65	86.33 ± 3.10	79.71 ± 3.58
Xenobiotics	94.82 ± 0.54	87.83 ± 2.15	83.95 ± 3.93	82.68 ± 1.30

Table 2: Class-wise performance analysis of multi-class GCN classifier

reviews, 111(9):5215–5246, 2011.

[3] S. Cohen, E. Ruppin, and G. Dror. Feature selection based on the shapley value. In *Proceedings of the 19th international joint conference on Artificial intelligence*, pages 665–670, 2005.

[4] B. M. El-Haj, S. B. Ahmed, M. A. Garawi, and H. S. Ali. Linking aromatic hydroxy metabolic functionalization of drug molecules to structure and pharmacologic activity. *Molecules*, 23(9):2119, 2018.

[5] T. J. Ewing, S. Makino, A. G. Skillman, and I. D. Kuntz. Dock 4.0: search strategies for automated molecular docking of flexible molecule databases. *Journal of computer-aided molecular design*, 15:411–428, 2001.

[6] G. Fuchs. Anaerobic metabolism of aromatic compounds. *Annals of the New York Academy of Sciences*, 1125(1):82–99, 2008.

[7] C. Hansch, W. E. Steinmetz, A. J. Leo, S. B. Mekapati, A. Kurup, and D. Hoekman. On the role of polarizability in chemical- biological interactions. *Journal of chemical information and computer sciences*, 43(1):120–125, 2003.

[8] A. J. Leo and C. Hansch. Role of hydrophobic effects in mechanistic qsar. *Perspectives in Drug Discovery and Design*, 17:1–25, 1999.

[9] C. S. Leung, S. S. Leung, J. Tirado-Rives, and W. L. Jorgensen. Methyl effects on protein–ligand binding. *Journal of medicinal chemistry*, 55(9):4489–4500, 2012.

[10] D. F. Lewis, M. N. Jacobs, and M. Dickins. Compound lipophilicity for substrate binding to human p450s in drug metabolism. *Drug discovery today*, 9(12):530–537, 2004.

[11] D. F. Lewis, B. G. Lake, and M. Dickins. Quantitative structure-activity relationships (qsars) in inhibitors of various cytochromes p450: The importance of compound lipophilicity. *journal of enzyme inhibition and medicinal chemistry*, 22(1):1–6, 2007.

[12] C. A. Lipinski, F. Lombardo, B. W. Dominy, and P. J. Feeney. Experimental and computational approaches to estimate solubility and permeability in drug discovery and development settings. *Advanced drug delivery reviews*, 23(1-3):3–25, 1997.

[13] S. A. Wildman and G. M. Crippen. Prediction of physicochemical parameters by atomic contributions. *Journal of chemical information and computer sciences*, 39(5):868–873, 1999.
